# Volume gain of periaqueductal gray in medication-overuse headache

**DOI:** 10.1186/s10194-016-0715-9

**Published:** 2017-02-01

**Authors:** Zhiye Chen, Xiaoyan Chen, Mengqi Liu, Shuangfeng Liu, Lin Ma, Shengyuan Yu

**Affiliations:** 1 Department of Radiology, Chinese PLA General Hospital, 28 Fuxing Road, Beijing, 100853 China; 2 Department of Neurology, Chinese PLA General Hospital, 28 Fuxing Road, Beijing, 100853 China

**Keywords:** Medication-overuse headache, Migraine, Periaqueductal gray, Magnetic resonance imaging

## Abstract

**Background:**

Periaqueductal gray (PAG) is a substantial descending pain modulatory center, and previous voxel-based morphometry study confirmed the clusters with increased volume in PAG region in medication-overuse headache (MOH). The aim of this study is to investigate altered PAG volume in MOH using an automated PAG segment method to measure the true PAG volume.

**Methods:**

High resolution three-dimensional T1-weighted fast spoiled gradient recalled echo MR images were obtained from 22 patients with MOH and 22 normal controls (NC). PAG template was created based on ICBM 152 gray template, and the individual PAG was generated by applying the deformation field from structural image segment to the PAG template, and individual PAG volume was calculated.

**Results:**

There was a significant increased volume of PAG in MOH (0.366 ± 0.005 ml) than that in NC (0.341 ± 0.005 ml)(*P* < 0.05). There was no significant correlation between the PAG volume and the clinical variables in MOH patients (*P* > 0.05). The area of receiver operating characteristic (ROC) curve was 0.845, and the cut-off of PAG volume was 0.341 ml with sensitivity 95.5% and specificity 63.6%.

**Conclusion:**

The present study demonstrated that the PAG volume gain was confirmed in MOH patients, and the automated individual PAG volume measure may be considered as a simple and effective imaging biomarker in MOH diagnosis.

## Background

Periaqueductal gray (PAG) was an anatomic and functional interface between forebrain and brainstem, and PAG was classified into 4 columns with different cytoarchitecture and included multiple types of neurons (eg. L-glutamate,γ-aminobutyric acid (GABA), opioids (particularly enkephalin), substance P) [1]. The PAG columns had distinct connections with the forebrain, brainstem, and nociceptive neurons of lamina I of the spinal cord and trigeminal nucleus [2–4]. PAG is a critical component of a network that is activated in response to pain, and the different PAG columns receive functionally segregated input from nociceptive pathways [5–7]. Previous studies demonstrated that PAG activation was modulated by expectation of pain [8] and placebo analgesia [9].

PAG is a substantial descending pain modulatory center, which exerts a dual control (including inhibition and facilitation) on nociceptive transmission in the dorsal horn and trigeminal nucleus [10], and the modulatory mechanism was exerted by descending PAG-RVM (rostral ventromedial medulla) pathway contributing to central sensitization and development of secondary hyperalgesia [10, 11]. PAG dysfunction was recognized in migraine [12], and functional MRI studies demonstrated that the PAG dysfunction was associated with increased iron deposition, which may play a role in the genesis or pathophysiology of MOH [13–15].

In the previous studies, the conventional MR imaging demonstrated that specific lesion such as multiple sclerosis and infarction in PAG may produced the migraine-like symptoms [16–21]. and nonspecific lesions without definite clinical diagnosis in PAG region [22]. Voxel-based morphometry (VBM) powerfully demonstrated that the clusters with PAG volume increase in MOH [23] and the clusters with PAG volume reducement in MOH with detoxification treatment response [24]. Therefore, PAG volume changes become an important imaging variable in MOH for the diagnosis and the treatment assessment.

VBM methods commonly could compare groups of individuals, and it also could be used to compare single case with control groups although a very high false positive rates presents [25] and non-parametric statistics could addressing the problem of high false positive rate in single case VBM [26]. As these methods were performed over the whole brain level, an automated individual PAG volume measurement was applied to MOH patients in the current study, and the expected cut-off value of PAG volume would be provided for the MOH diagnosis.

## Methods

### Subjects

Written informed consent was obtained from all participants according to the approval of the ethics committee of the local institutional review board. Twenty-two MOH patients were recruited from the International Headache Center, Department of Neurology, Chinese PLA General Hospital. All the following inclusion criteria should be fulfilled: 1) diagnosis of 8.2 MOH, and 1.1 and 1.2 migraine based on the International Classification of Headache Disorders, third Edition (beta version) (ICHD-III beta) [27]; 2) no migraine preventive medication used in the past 3 months; 3) age between 20 and 60 years; 4) right-handed; 5) patient’s willingness to engage in the study. The exclusion criteria were the following: 1) with any chronic disorders, including hypertension, hypercholesterolemia, diabetes mellitus, cardiovascular diseases, cerebrovascular disorders, neoplastic diseases, infectious diseases, connective tissue diseases, other subtypes of headache, chronic pain other than headache, severe anxiety or depression preceding the onset of headache, psychiatric diseases, etc.; 2) with alcohol, nicotine, or other substance abuse; 3) with cranium trauma, illness interfering with central nervous system function, psychotic disorder, and regular use of a psychoactive or hormone medication. Twenty-two normal controls (NCs) were recruited from the hospital’s staff and their relatives. Inclusion criteria were similar to those of patients, except for the first two items. NCs should never have had any primary headache disorders or other types of headache in the past year. General demographic and headache information were registered and evaluated in our headache database. Additionally, we evaluated anxiety, depression, and cognitive function of all the participants by using the Hamilton Anxiety Scale (HAMA) [28], the Hamilton Depression Scale (HAMD) [29], and the Montreal Cognitive Assessment (MoCA) Beijing Version (www.mocatest.org). The study protocol was approved by the Ethical Committee of Chinese PLA General Hospital and complied with the Declaration of Helsinki. Informed consent was obtained from all participants before the study. MRI scans were taken in the interictal stage at least three days after a migraine attack for MOH patients. All the patients were given with the Visual Analogue Scale (VAS). All the subjects were right-handed and underwent conventional MRI examination to exclude the subjects with cerebral infarction, malacia, or occupying lesions. Alcohol, nicotine, caffeine, and other substances were avoided for at least 12 h before MRI examination.

### MRI acquisition

Images were acquired on a GE 3.0 T MR system (DISCOVERY MR750, GE Healthcare, Milwaukee, WI, USA) and a conventional eight-channel quadrature head coil was used. All subjects were instructed to lie in a supine position, and formed padding was used to limit head movement. A three-dimensional T1-weighted fast spoiled gradient recalled echo (3D T1-FSPGR) sequence generating 180 contiguous axial slices [TR (repetition time) = 6.3 ms, TE (echo time) = 2.8 ms, flip angle = 15o, FOV (field of view) = 25.6 cm × 25.6 cm, Matrix = 256 × 256, NEX (number of acquisition) = 1] was used to perform the new segment and the individual PAG creation. Conventional T2-weighted imaging (T2WI) and T1 fluid-attenuated inversion recovery (T1-FLAIR) weighted imaging were also acquired. All imaging protocols were identical for all subjects. No obvious structural damage and T2-visible lesion were observed based on the conventional MR images.

### MR image processing

**Fig. 1:**
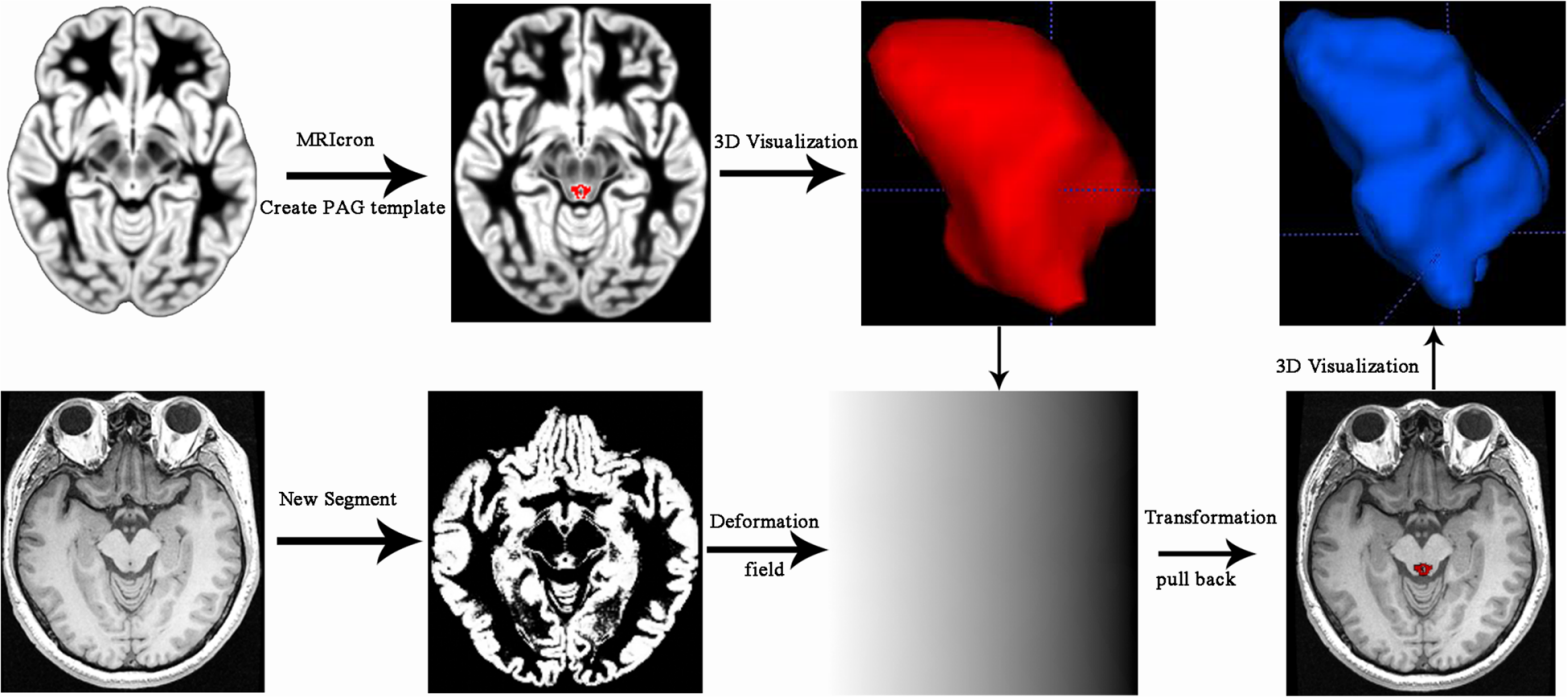
The flow chart of PAG template and individual PAG segmentation. Top line: PAG template was created using MRIcron software based standard MNI template (left 3 columns); Bottom line: Individual PAG segment was performed using New segment tool (SPM8 software) and then applied the generated deformation field to PAG template to generate individual PAG. Red and blue represent 3D visualization of PAG template and individual PAG

All MR structural image data were processed using Statistical Parametric Mapping 12 (SPM 12) (http://www.fil.ion.ucl.ac.uk/spm/) running under MATLAB 7.6 (The Mathworks, Natick, MA, USA) to perform segment [30]. The image processing included following steps: (1) Create PAG template based on mni_icbm152_gm_tal_nlin_asym_09a template by pen tool using MRIcron software (www.mricro.com); (2) Individual PAG segment by apply the deformation field (generated by segment) to the PAG template using the runback strategy; (3) calculate the individual PAG volume using ITK-SNAP (V3.6.0-beta) (http://www.itksnap.org/pmwiki/pmwiki.php) (Fig. 1).

### Statistical analysis

The statistical analysis was performed by using PASW Statistics 18.0. The significance differences of PAG volume between MOH group and NC group were computed using general linear model (independent univariate *t*-test with age and sex as covariates). The Pearson's correlation analysis was applied between PAG volume and the clinical variables in MOH. The age, HAMA, HAMD and MoCA were performed with independent samples *T* test, and sex was performed with Chi-Square test. Significant difference was set at a P value of < 0.05. Receiver operating characteristic (ROC) curve analysis was performed to identify the diagnostic efficacy and the cut-off value of PAG volume for MOH.

## Results

### Demography and neuropsychological test

Twenty-two MOH patient (F/M = 14/8) and 22 NCs (F/M = 14/4) were enrolled (Table 1). There was no significant difference for age and sex between MOH (42.55 ± 10.31 years old) and NC (45.09 ± 10.50 years old). There was significant difference for the scores of HAMA, HAMD and MoCA in MOH compared with that in NC (*P* < 0.05). The disease duration was 6.65 ± 8.00 years, and the scores of VAS were 8.27 ± 1.52. The type of overused medication in MOH patients included:simple analgesics (3/22), simple triptans (1/22), and combination analgesics (18/22).

**Table 1:** The clinical characteristics of MOH patients and normal controls(NC)

	MOH	NC	T value	*P* value
Num(F/M)	22(14/8)	22(19/3)	3.03^a^	0.16
Age(yrs)	42.55 ± 10.31	45.09 ± 10.50	0.81	0.42
HAMA	15.82 ± 6.81	9.77 ± 3.15	3.78	0.00
HAMD	17.91 ± 8.88	8.09 ± 4.80	4.54	0.00
MoCA	24.23 ± 3.21	27.50 ± 1.97	4.08	0.00
DD(yrs)	6.65 ± 8.00	NA	NA	NA
VAS	8.27 ± 1.52	NA	NA	NA

*NA* Not available, *DD* disease duration

^a^Chi-Square test

### Comparison of PAG volume between MOH group and NC group

**Fig. 2:**
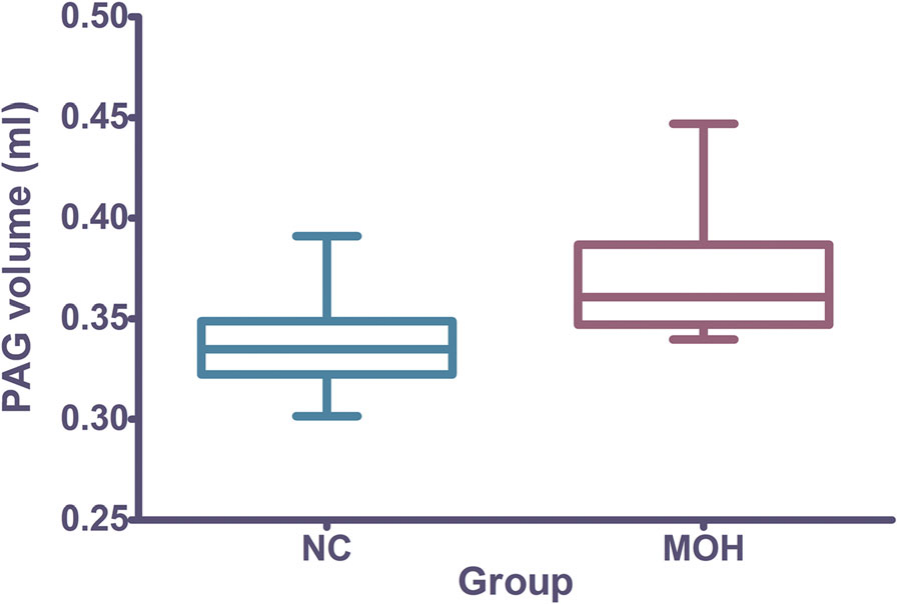
PAG volume distribution plot of MOH and NC

There was a significant increased volume of PAG in MOH (0.366 ± 0.005 ml) than that in NC (0.341 ± 0.005 ml)(*F* value = 14.41, *P* value = 0.000) (Fig. 2). There was no significant correlation between the PAG volume and the clinical variables in MOH patients (*P* > 0.05).

### ROC analysis between MOH group and NC group

**Fig. 3:**
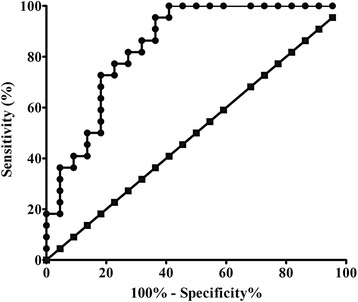
ROC curve of PAG volume for the diagnosis of MOH from NC. The area under the curve is 0.845

The area of receiver operating characteristic (ROC) curve was 0.845, and the cut-off of PAG volume was 0.341 ml with sensitivity 95.5% and specificity 63.6% (Fig. 3).

## Discussion

PAG dysfunction had been identified in MOH patients, and the investigation methods including resting-state functional MRI [31–33], diffusion kurtosis imaging [34], positron emission tomography [35], and iron deposition study [13]. The altered PAG function [33] may result in the change of PAG volume. The previous VBM study identified clusters in PAG region with volume increase [36], which was based on normalized grey matter. However, the current study provided the true individual PAG volume change firstly in vivo.

The present findings demonstrated the PAG volume gain in MOH compared with NC, which may explain the PAG dysfunction in MOH from the PAG structural pathophysiological change viewpoint. However, the correlation analysis showed that there was no any significant correlation between the PAG volume and the clinical variables in MOH patients. These findings suggested that PAG volume gain may only be associated with the descending pain modulatory network, and the other clinical factor such as anxiety, depress and cognitive function could not influence the PAG structural change in MOH although anxiety scores, depression scores showed significant difference between MOH and NC. The previous study [37] indicated that anxiety and depression scores was correlated with gray matter volumes of some cerebral regions, therefore, it could assume that anxiety and depression might impair the cerebral regions, and PAG volume gain might be specific change in MOH patients.

Although PAG volume gain was identified in MOH in this study, Fig. 2 present a small overlap between MOH and NC for PAG volume. Further ROC analysis demonstrated PAG volume measurement showed a good level for the diagnosis of MOH from NC. The cut-off value of PAG volume (0.341 ml) could provide a high sensitivity (95.5%), however, the specificity (63.6%) was relative. Therefore, it should be careful to explain the diagnosis of MOH when PAG volume around the cut-off value.

Compared with VBM study, the current study employed an automated PAG volume measurement. VBM study was generally used to compare the difference between groups, and individual PAG volume could not be measured by this method. In this study, the individual PAG segment was generated by applying the deformation filed to the PAG template, which also be recognized as pull back strategy. This automated PAG measurement method could be performed in the routine clinical practice, and had a widespread application for MOH diagnosis, even for the treatment response assessment. However, it should be more prudent when PAG volume was considered as a biomarker for the diagnosis of MOH since MOH is likely to remain a clinical diagnosis.

The limits for this study included as follows: (1) The sample was relative small, and the large sample of MOH and NC should be needed in the future study; (2) The present study was a cross-sectional study, and the longitudinal observation and the treatment response evaluation should further be performed in future study.

## Conclusion

In conclusion, PAG volume gain was identified in MOH patients, and PAG volume may be considered as an imaging biomarker for the diagnosis of MOH.

## Abbreviations

MOH: Medication-overuse headache
NC: Normal controls
PAG: Periaqueductal gray.
